# Intralesional 5-Fluorouracil in the Treatment of Squamous Cell Carcinoma in an Elderly Patient

**DOI:** 10.7759/cureus.55855

**Published:** 2024-03-09

**Authors:** Stephanie M McDonald, Peter J Neidenbach

**Affiliations:** 1 Department of Dermatology, Edward Via College of Osteopathic Medicine, Spartanburg, USA

**Keywords:** squamous cell carcinoma (scc), cutaneous squamous cell carcinoma (cscc), 5-fluorouracil, nonmelanoma skin cancer, 5-fu in squamous cell carcinoma, 5-fluorouracil in squamous cell carcinoma, intralesional 5-fluorouracil

## Abstract

Squamous cell carcinoma (SCC) is the second leading form of skin cancer. In the elderly population, surgery may carry more risk and significant morbidity in comparison to less invasive forms of treatment. This case report describes the successful use of intralesional 5-fluorouracil (IL5-FU) to treat cutaneous squamous cell carcinoma (cSCC). A 98-year-old white woman presented in early May 2017 with a 3.5-cm rapidly growing crusted nodule on her left proximal-lateral arm. She had a past medical history of chronic obstructive pulmonary disease, atrial fibrillation, and heart failure. The patient also had a frail body habitus and weighed 80 pounds. Physical examination revealed a large, ulcerated, crateriform mass on the left proximal-lateral arm. A shave biopsy was performed, which revealed a well-differentiated SCC, composed of nodular masses of neoplastic squamous cells with atypical nuclei, keratin pearl formation, and scattered mitotic figures with surrounding fibrosis and inflammation. The patient was wheelchair-bound and oxygen-dependent and, thus, not considered a good surgical or radiation candidate. Treatment was decided with 5-fluorouracil. At a four-week follow-up appointment, there was no visible or palpable evidence of the tumor. There was no sign of recurrence at three months, indicating treatment success. The patient later died due to cardiac arrest in September 2017. The elderly population with cSCC can benefit from intervention and treatment with IL5-FU when surgery is not an option due to patient comorbidities. IL5-FU can potentially be used in areas where access to a dermatologist, surgeon, or surgical services is limited.

## Introduction

Squamous cell carcinoma (SCC) is the second leading form of skin cancer [1]. In the elderly population, surgery may carry more risk and significant morbidity in comparison to less invasive forms of treatment. This case report describes the successful use of intralesional 5-fluorouracil (IL5-FU) to treat cutaneous squamous cell carcinoma (cSCC).

## Case presentation

Report of case

**Figure 1:**
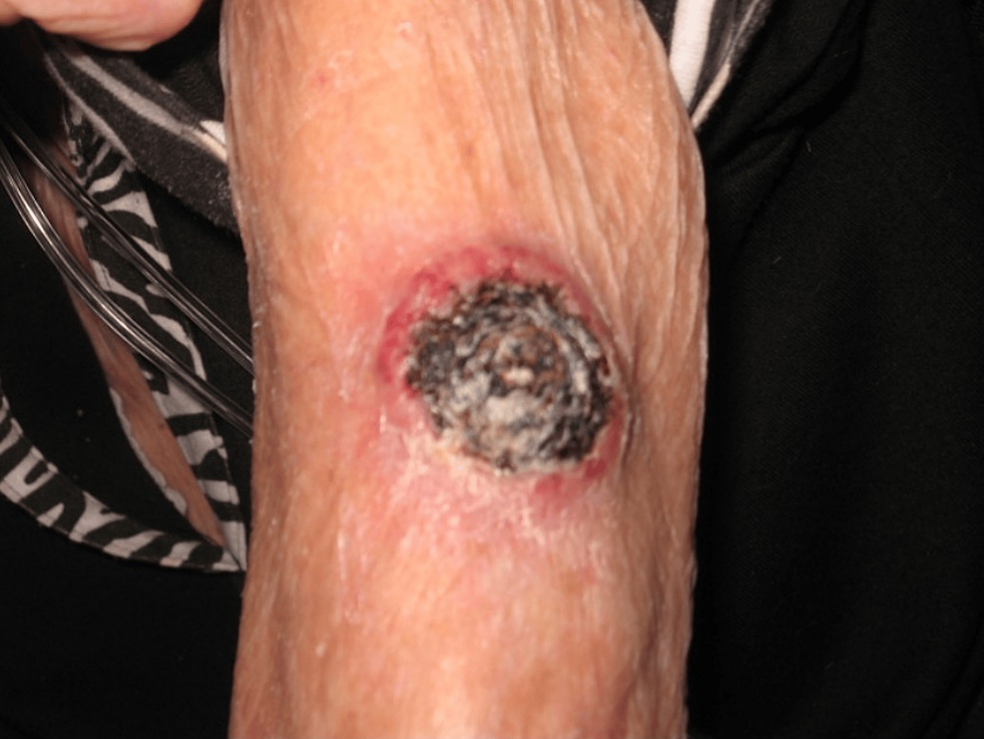
A large ulcerated, crateriform mass on the left proximal-lateral arm.

**Figure 2:**
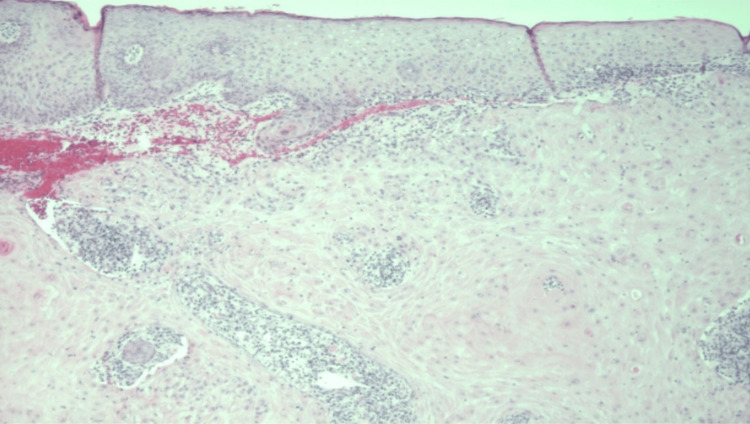
Squamous cell carcinoma, well-differentiated at 40X magnification. Stained using H&E stain. The tumor is composed of nodular masses of neoplastic squamous cells with atypical nuclei, keratin pearl formation, and scattered mitotic figures with surrounding fibrosis and inflammation present. H&E, hematoxylin and eosin

A 98-year-old white woman presented in early May 2017 with a 3.5-cm rapidly growing crusted nodule on her left proximal-lateral arm. She had a past medical history of chronic obstructive pulmonary disease, atrial fibrillation, and heart failure. The patient also had a frail body habitus and weighed 80 pounds. Physical examination revealed a large, ulcerated, crateriform mass on the left proximal-lateral arm (Figure 1). A shave biopsy was performed, which revealed a well-differentiated SCC, composed of nodular masses of neoplastic squamous cells with atypical nuclei, keratin pearl formation, and scattered mitotic figures with surrounding fibrosis and inflammation (Figure 2). The patient was wheelchair-bound and oxygen-dependent and, thus, not considered a good surgical or radiation candidate.

Diagnosis

A diagnosis of well-differentiated SCC was made.

Methods

The patient was deemed competent and had good cognitive function to provide verbal informed consent, which was obtained. After providing the patient with the risks versus benefits of the injection, treatment was decided with 5-fluorouracil (5-FU). The patient was prepared for the procedure with 1 cc of triamcinolone (Kenalog®, Bristol-Myers Squibb, Princeton, NJ) 40 mg/mL diluted to 3 cc with bupivacaine hydrochloride (Marcaine, Pfizer, New York, NY) 0.5%. These were injected circumferentially. Following this injection, 1.5 cc of 5-FU 50 mg/mL solution was injected peripherally into the buttress of the tumor, also known as an intralesional injection. Petrolatum ointment (Aquaphor, Beiersdorf, Hamburg, Germany), non-adherent dressing pads (Telfa, Cardinal Health, Dublin, OH), and self-adherent wrap (Coban, 3M, St. Paul, MN) with latex wrap were applied and left on overnight.

Results

**Figure 3:**
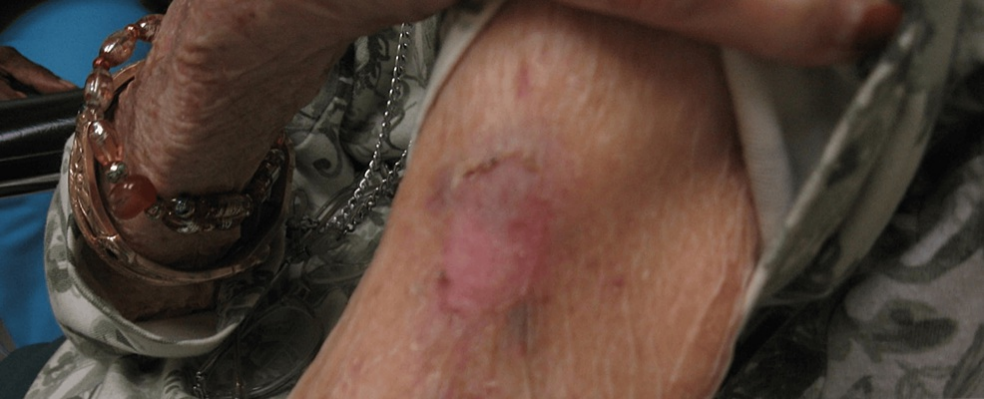
Follow-up status at four weeks post-intralesional 5-FU injection.

**Figure 4:**
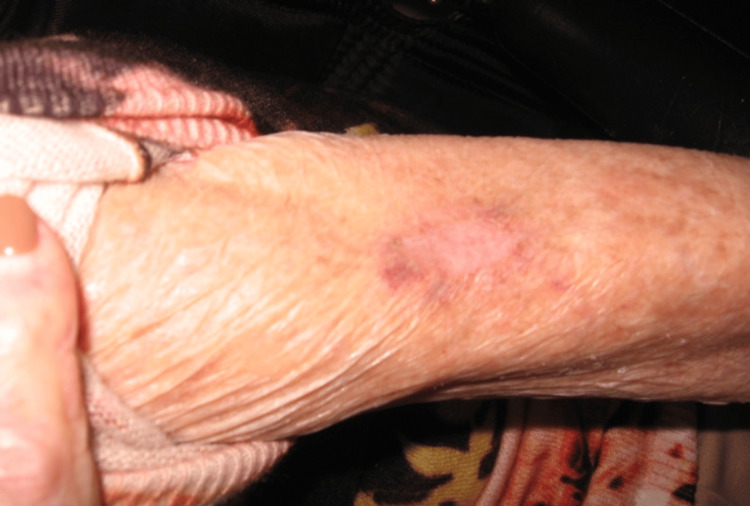
Result at three months. No lesion present post-intralesional 5-FU injection.

At the four-week follow-up appointment, there was no visible or palpable evidence of the tumor (Figure 3). There was no sign of recurrence at three months, indicating treatment success (Figure 4). The patient later died due to cardiac arrest in September 2017.

## Discussion

cSCC tends to arise after UV exposure due to induced DNA damage [1]. To prevent SCC, limiting sun exposure and wearing sunscreen of a minimum of SPF 30 are essential to reduce one’s risk. SCC develops from malignant proliferation of the squamous cells in the skin (keratinocytes). The precancerous lesion of SCC is actinic keratosis. Mutations in the tumor suppressor gene TP53 have been elucidated as an early event that occurs after sun exposure [1]. SCCs have metastatic potential if not treated.

Furthermore, 5-FU is a chemotherapy agent that works as an antimetabolite-pyrimidine analog that blocks thymidylate synthetase, which, in turn, blocks DNA synthesis [2]. This drug specifically is converted to fluorodeoxyuridine monophosphate (FdUMP), which binds with thymidylate synthase to block the production of deoxythymidine monophosphate (dTMP) or thymidylate. dTMP is needed for DNA repair; 5-FU causes significant damage to dTMP’s ability to repair DNA [3,4]. This agent is currently FDA-approved for various cancers, and it has been particularly beneficial in the topical treatment of other dermatologic neoplasia such as basal cell carcinomas [3,5].

The gold standard of care in the treatment of SCC is surgical removal [3]. This poses a problem in patients who are unable to tolerate surgery and/or the associated complications with surgery. Complications of cutaneous surgery in an elderly patient or a patient with comorbidities include improper wound healing, the risk of infection, excessive bleeding, pain, and the development of a larger scar. The elderly population may be at a greater risk of improper wound healing due to factors such as malnutrition, stress, certain medications, and comorbid diseases that affect blood circulation such as peripheral vascular disease and chronic venous insufficiency [6]. For example, Mohs micrographic surgery can lead to a wound that does not heal properly due to infection and/or other comorbidities [7]. These stated risks were reduced via the minimally invasive injections of IL5-FU in comparison to more invasive surgery. Thus, there is low morbidity associated with this form of treatment.

In this case report, the patient was frail with thin skin and comorbidities. The patient was wheelchair-bound and oxygen-dependent and, thus, not considered a good surgical candidate nor a candidate for radiation. The older population can benefit from intervention and treatment of SCC with IL5-FU. IL5-FU can potentially be used in areas where access to a dermatologist, surgeon, or surgical services is limited. Studies have demonstrated cure rates between 92% and 95% [3,8,9]. Moreover, regarding cost, IL5-FU is more cost-effective in comparison to surgery. A vial of 5-FU costs anywhere from $19.50 to $26, whereas the cost of a wide local excision averages $4,699.41 and the cost of Mohs micrographic surgery averages $4,365.50 [3,10].

While there are great benefits to this treatment, it is important to discuss the limitations and adverse effects of using IL5-FU. A limitation for patients obtaining this form of treatment includes the lack of a standard dose for the volume of the injection and a standard preparation procedure for the injection. Guidelines currently do not mention or support the use of IL5-FU in the treatment of cSCC, and the volume amount of IL5-FU has not been standardized [8]. The guidelines mention the use of a topical cream, also known as Efudex, but the guidelines do not mention the use of IL5-FU [8]. There have been only a few mentions of IL5-FU in the literature. A retrospective cohort study was conducted by identifying 148 patients with cSCCs and the use of IL5-FU along with 1% lidocaine with 1:100,000 epinephrine for local anesthesia [9]. This study used a different preparation to anesthetize the area. The volume of 5-FU varied from 0.2 mL to 2 mL for each lesion, indicating that this treatment option has not yet been standardized [9]. The size of the lesion may also play a role in determining the volume of the 5-FU injection. Furthermore, in a case report by Vazquez et al., a 96-year-old male presented with a cSCC on the lower extremity and was treated with a similar method to our patient using lidocaine for the anesthetic and multiple injections of 5-FU 50 mg/mL solution for 1.5 mL in total [11]. Compared to the 98-year-old female patient in our case report, this was the same amount of 5-FU. However, it is to be noted that lidocaine was the anesthetic, and trichloroacetic acid (TCA) was subsequently used in the 96-year-old male patient. Although the male patient saw complete regression of the tumor with the use of IL5-FU, the use of TCA may pose a confounding variable in studying the effect of IL5-FU [11].

Another limitation for patients is the lack of access to a dermatologist. Access to a dermatologist is difficult to obtain for patients, such as those in rural areas, and there are non-compliance issues due to the distance from the nearest dermatologist [12]. Primary care physicians (PCPs) could potentially use IL5-FU areas where access is limited. These PCPs could obtain training through a workshop on how to evaluate SCCs in the primary care setting and how to use IL5-FU to treat appropriate patients. However, this poses the following questions: “Who is going to create the learning material for the physicians?” “Who exactly would train these PCPs?” “How many of these physicians feel comfortable treating SCCs in their office?” and “How can we be sure that there are clear margins if a pathologist is not readily available?” A survey can be completed to gain insight into how PCPs feel about treating SCCs successfully in the office. Adverse effects of IL5-FU, including ulceration, stinging, and burning during the injection, erythema, swelling, discomfort during the injection, tenderness, edema, eschar, depressed scar caused by the injection, crusting, and erosion, have been reported [3].

A randomized controlled trial should be conducted to compare surgery versus the injection of IL5-FU on a larger number of patients with cSCC to determine the maximum effective dose of IL5-FU, to create a standard preparation procedure for the injection and aftercare, and to confirm the efficacy of IL5-FU. Furthermore, it should not be used in patients with metastasis of SCC to the lymph node.

## Conclusions

The older population with cSCC can benefit from intervention and treatment with IL5-FU when surgery is not an option due to patient comorbidities. IL5-FU can potentially be used in areas where access to a dermatologist, surgeon, or access to surgical services is limited.

## References

[1] Kumar V, Abbas AK, Aster JC (2017). The skin. Robbins Basic Pathology.

[2] (2024). Fluorouracil, 5-FU. https://www.clinicalkey.com/#!/content/drug_monograph/6-s2.0-258?scrollTo=%23Indications.

[3] Metterle L, Nelson C, Patel N (2016). Intralesional 5-fluorouracil (FU) as a treatment for nonmelanoma skin cancer (NMSC): a review. J Am Acad Dermatol.

[4] Zhang N, Yin Y, Xu SJ, Chen WS (2008). 5-Fluorouracil: mechanisms of resistance and reversal strategies. Molecules.

[5] Kirby JS, Miller CJ (2010). Intralesional chemotherapy for nonmelanoma skin cancer: a practical review. J Am Acad Dermatol.

[6] Sgonc R, Gruber J (2013). Age-related aspects of cutaneous wound healing: a mini-review. Gerontology.

[7] Alam M, Ibrahim O, Nodzenski M (2013). Adverse events associated with mohs micrographic surgery: multicenter prospective cohort study of 20,821 cases at 23 centers. JAMA Dermatol.

[8] Kim JY, Kozlow JH, Mittal B, Moyer J, Olenecki T, Rodgers P (2018). Guidelines of care for the management of cutaneous squamous cell carcinoma. J Am Acad Dermatol.

[9] Maxfield L, Shah M, Schwartz C, Tanner LS, Appel J (2021). Intralesional 5-fluorouracil for the treatment of squamous cell carcinomas. J Am Acad Dermatol.

[10] Udkoff J, Beal BT, Brodland DG, Knackstedt T (2022). Cost effectiveness of intermediate-risk squamous cell carcinoma treated with Mohs micrographic surgery compared with wide local excision. J Am Acad Dermatol.

[11] Vazquez T, Chalela JG, Florez-White Florez-White, M M (2019). A patient with squamous cell carcinoma in-situ successfully treated with intralesional 5-FU and topical TCA. J Am Acad Dermatol.

[12] Asbeck SM, Imo BU, Okobi OE, Dorcé-Medard J (2023). The dermatologic care needs of a rural community in South Florida. Int J Environ Res Public Health.

